# Rooting through single-cell sequencing in phloem pole cells

**DOI:** 10.1038/s42003-022-04180-4

**Published:** 2022-11-07

**Authors:** Jinbao Liu, Aqsa Majeed, M. Shahid Mukhtar

**Affiliations:** Department of Biology, University of Alabama at Birmingham, Birmingham, AL 35294 USA

## Abstract

While recent advances in plant single-cell RNA sequencing (scRNA-seq) have made numerous strides in identifying novel regulatory events, transcriptional profiling of certain cell types, such as phloem poles, has not yet been thoroughly investigated. A recent article by Otero et al. utilized cell-type specific marker lines and a second-generation single-cell approach to uncover transcriptomic landscapes specifying protophloem-adjacent cells, as well as identify a set of important transcription factors (TFs) signifying early phloem development.

Plant organogenesis involves extensive spatiotemporal coordination among diverse cell populations, each with distinct transcriptional signatures that contribute to their developmental trajectories and organ development. Single-cell RNA sequencing (scRNA-seq) has established high-resolution cell-type specific atlases that provide insight into both shared and distinctive transcriptome signatures across diverse cell types in developing plants^1,2^. For instance, Shahan et al. recently constructed an organ-scale single-cell transcriptomic map in *Arabidopsis* root^2^. However, such a global approach may not completely capture the intricate gene regulation carried out by cell subpopulations that are spatially adjacent but undergoing distinct differentiation programs, owing to the inadequate sequencing resolution in specific root areas. For instance, phloem poles, structures involved in long-distance transport of sugar and other metabolites, are characterized by four heterogeneous cell types: protophloem sieve elements, metaphloem sieve elements, phloem pole pericycles and companion cells. As most of these cell types play an important role in phloem pole development, they were found underrepresented in previous scRNA-seq studies. To deconvolute this heterogeneity, Otero et al.^3^ combined cell-type specific marker lines, fluorescence-activated cell sorting and SMRT-sequencing technologies to obtain high-resolution scRNA-seq profiles within protophloem sieve element-neighboring regions across developmental stages^3^ (Fig. 1). The authors successfully sorted and enriched cell types based on distinct fluorescent translational fusion lines, including sieve element, companion cell and phloem pole pericycle, at a continuity of developmental stages.

**Fig. 1:**
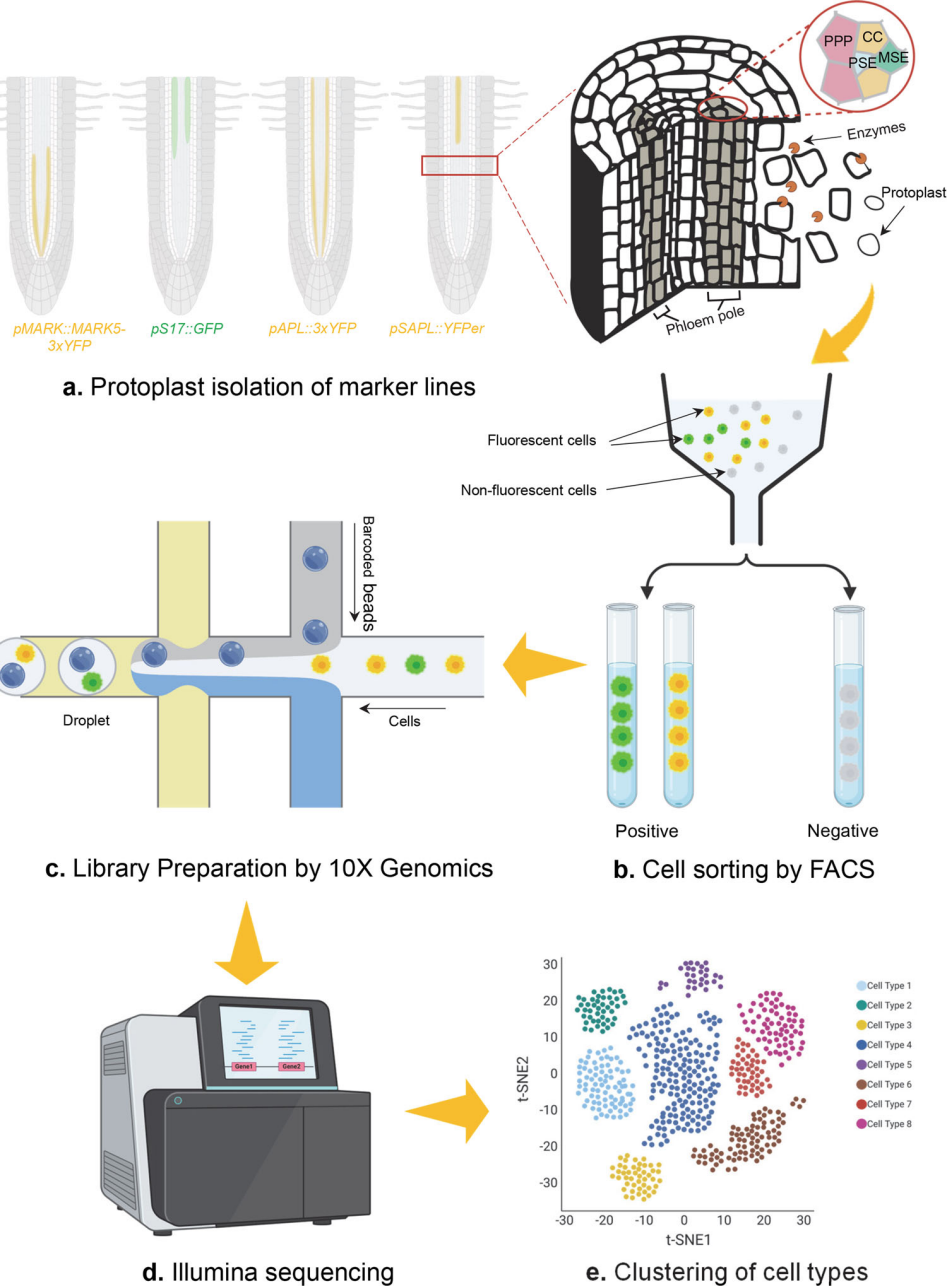
Workflow of second-generation scRNA-seq. Cells derived from marker lines specifying cell identities are dissociated via protoplast isolation (**a**), followed by fluorescence-activated cell sorting (FACS) to enrich target positive cells and eliminate unwanted negative cells (**b**). Positive cells collected are later used to prepare cDNA library within individual droplet (**c**) and subsequently got sequenced (**d**). Based on transcriptomic data coming from single cells, cell identities and development atlas are finally profiled (**e**).

Subsequently, the authors developed a high-quality scRNA-seq dataset encompassing 10,204 phloem pole cells, from which they inferred 15 representative clusters with diverse cell identities and developmental statuses^3^. Developmental trajectories analysis further resolved clusters representing early, undifferentiated stages of cells that were prone to three different cell fates: metaphloem sieve element (cluster 8), phloem pole pericycle (cluster 9), and companion cell (cluster 10). Interestingly, these protophloem sieve element-adjacent cells show similar co-expression patterns even after differentiation, leading to a better understanding of how protophloem sieve elements rely on their surroundings before and after enucleation^3^. The authors also managed to identify early metaphloem sieve elements in clusters 10 and 1, suggesting the existence of intertwining cell fates during phloem morphogenesis^3^. Finally, a group of TFs pertaining to the DNA-binding with one finger (DOF) family, PINEAPPLEs (PAPLs), were validated as downstream targets of *PHLOEM EARLY DOF* (*PEAR*) genes in early phloem. PAPLs appear to be of critical importance to sugar transport into root, but further study is needed to characterize their precise contributions to nutrient allocation^3^.

In summary, this study provides a broad perspective of the developmental trajectories of the phloem poles as well as highlights the critical importance of DOF TFs to phloem development^3^. This study also outlines a technical framework that can potentially be extended to other plant organs to determine spatiotemporal gene regulation both at steady state and under diverse environmental conditions.
